# Modifiable Factors Influencing Emotional Intelligence Among Medical Interns

**DOI:** 10.1192/bjo.2022.139

**Published:** 2022-06-20

**Authors:** Nurulhuda Mat Hassan, Norwati Daud, Nik Nor Ronaidi Nik Mahdi, Mohd Salami Ibrahim, Yuzana Mohd Yusop, Mohd Faeiz Pauzi

**Affiliations:** Universiti Sultan Zainal Abidin, Kuala Terengganu, Malaysia

## Abstract

**Aims:**

Emotional intelligence is crucial for medical professionals. Medical interns are expected to have a high degree of emotional intelligence to face their professional career challenges. Emotional intelligence, often measured as an emotional quotient (EQ), is the capacity to recognize and regulate emotion in oneself. It enables one to monitor own feelings and emotions and others; and guide decisions and actions, and is crucial to ensure a successful work-related outcome or good performance. A higher EQ enhances physician and patient well-being, increases patient safety and augments healthcare teamwork. However, studies about EQ among medical interns are lacking. Therefore, this study intended to determine the level of EQ among medical interns in Malaysia and its associated factors.

**Methods:**

This nationwide cross-sectional study recruited new medical interns reporting to 17 randomly selected Malaysian hospitals accredited for medical intern training from January to April 2020. They were invited to answer an online questionnaire incorporating USMEQ-i to measure EQ, Connor-Davidson Resilience Scale-10 items (CD-RISC-10) for resilience, Brief-Cope to assess coping styles, PHPQ to assess internship preparedness, DUREL for religiosity, and questions related to sociodemographic and undergraduate training.

**Results:**

A total of 524 from 619 medical interns responded. Mean (SD) EQ score was 3.08(0.58). Significant factors positively associated with EQ include resilience score (adjusted b = 0.65, 95% CI 0.58, 0.72, p <0.001), preparedness for internship (adjusted b = 0.11, 95% CI 0.09, 0.13, p < 0.001), approach-style coping (adjusted b = 0.17, 95% CI 0.11, 0.24, p <0.001), and religiosity (adjusted b = 0.09, 95% CI 0.01, 0.17, p <0.001). In contrast, avoidant-style coping (adjusted b=−0.19, 95% CI –0.28, 0.11, p <0.001) is negatively associated with EQ. Adjusted R2 of 67.6% substantiated the goodness of fit of the regression model.

**Conclusion:**

This study showed that there are a few modifiable factors that significantly influence EQ among medical interns; namely resilience, coping style, preparedness for internship, and religiosity. There is a positive association between EQ and approach coping style, and a negative relationship with avoidant coping. Approach coping encapsulates constructive responses to stress such as positive reframing, acceptance, seeking helpful information, and reaching for emotional support, while avoidant coping includes self-distraction, denial, venting, substance abuse, behavioural disengagement, and self-blame. These significant factors in this study such as coping and resilience can be learned and taught as a skill. These findings will aid medical schools to design programmes and improve the medical education to increase EQ among medical students who will become better medical interns and doctors in the future.

